# DeepVinci: Organ and Tool Segmentation with Edge Supervision and a Densely Multi-Scale Pyramid Module for Robot-Assisted Surgery

**DOI:** 10.3390/diagnostics15151917

**Published:** 2025-07-30

**Authors:** Li-An Tseng, Yuan-Chih Tsai, Meng-Yi Bai, Mei-Fang Li, Yi-Liang Lee, Kai-Jo Chiang, Yu-Chi Wang, Jing-Ming Guo

**Affiliations:** 1 Department of Electrical Engineering, National Taiwan University of Science and Technology, Taipei 106335, Taiwan; sfgx8801234@gmail.com; 2 Graduate Institute of Applied Science and Technology, National Taiwan University of Science and Technology, Taipei 106335, Taiwan; yuanchihtsai@gmail.com; 3 Graduate Institute of Biomedical Engineering, National Taiwan University of Science and Technology, Taipei 10607, Taiwan; 4 Adjunct Appointment to Department of Biomedical Engineering, National Defense Medical Center, Taipei 11490, Taiwan; 5 Department of Obstetrics and Gynecology, Tri-Service General Hospital, National Defense Medical Center, Taipei 114202, Taiwan; 6 Department of Obstetrics and Gynecology, Kang-Ning General Hospital, Kang Ning University, Taipei 114046, Taiwan; 7 Department of Nursing, Tri-Service General Hospital, National Defense Medical Center, Taipei 114, Taiwan; 8 School of Nursing, National Defense Medical Center, Taipei 114, Taiwan

**Keywords:** artificial intelligence, da Vinci Robot, gynecological surgery, organ semantic segmentation, deep learning

## Abstract

**Background**: Automated surgical navigation can be separated into three stages: (1) organ identification and localization, (2) identification of the organs requiring further surgery, and (3) automated planning of the operation path and steps. With its ideal visual and operating system, the da Vinci surgical system provides a promising platform for automated surgical navigation. This study focuses on the first step in automated surgical navigation by identifying organs in gynecological surgery. **Methods**: Due to the difficulty of collecting da Vinci gynecological endoscopy data, we propose DeepVinci, a novel end-to-end high-performance encoder–decoder network based on convolutional neural networks (CNNs) for pixel-level organ semantic segmentation. Specifically, to overcome the drawback of a limited field of view, we incorporate a densely multi-scale pyramid module and feature fusion module, which can also enhance the global context information. In addition, the system integrates an edge supervision network to refine the segmented results on the decoding side. **Results**: Experimental results show that DeepVinci can achieve state-of-the-art accuracy, obtaining dice similarity coefficient and mean pixel accuracy values of 0.684 and 0.700, respectively. **Conclusions**: The proposed DeepVinci network presents a practical and competitive semantic segmentation solution for da Vinci gynecological surgery.

## 1. Introduction

With advances in both software and hardware, the use of artificial intelligence (AI) has spread in daily life. These advances have led to the development of AI medical assistance technologies such as brain tumor segmentation, stroke prediction, and polyp status recognition. However, challenges still remain for achieving fully automated surgical navigation.

In the field of gynecology, common surgical cases include uterine myoma and benign ovarian tumors, with uterine myoma being the most common uterine tumor. Approximately 20–30% of women over the age of 35 years have this condition; however, women are only aware that they have this condition if there is pain or bleeding. Surgery is performed if medical treatment has failed or if there is a possibility of malignancy. Since Harry Reich first proposed laparoscopic hysterectomy in 1988, minimally invasive surgery has become the most prevalent approach for benign gynecologic surgery [1]. Laparoscopic surgery enables enhanced visualization of the surgical field, leading to smaller surgical wounds, less blood loss, and reduced postoperative pain [2].

Robotic surgery, which can be applied to minimally invasive surgery, was first performed in 1985 for neurosurgical biopsy [3]. Currently, the da Vinci Robot, developed by Intuitive Surgical, Inc. (Sunnyvale, CA, USA), is used worldwide for various surgeries, including malignant gynecologic surgery. The da Vinci Robot consists of one or two consoles, the Tilepro system, and the Firefly system. The field is clear and three-dimensional, and the operator can remotely control the arms in the console. Moreover, the range of motion of the robotic arm is less restricted than that of a laparoscope [4].

Deep learning, which simulates the operation of human neural networks, is a rapidly growing technology with great potential to enhance robot-assisted surgery. Common deep learning architectures include multilayer perceptrons, convolutional neural networks (CNNs), recurrent neural networks, and long short-term memory networks. Deep learning has been shown to be more effective than traditional rule-based approaches and is particularly applicable to visual recognition, natural language processing, and biomedicine.

In robot-assisted surgery, deep learning is used to detect robotic arms with surgical instruments. For example, Sarikaya et al. proposed multimodal CNNs to detect tools in the Fundamental Skills of Robotic Surgery curriculum [5]. Using the Faster R-CNN, Jin et al. successfully detected various instruments in laparoscopic cholecystectomies, such as grasper, bipolar, and clipper instruments [6]. Yamazaki et al. used the YOLOv3 algorithm to detect electric cautery and energy devices in laparoscopic gastrectomy [7]. Ibn Azad et al. detected tumors using the YOLOv4 algorithm and classification with gradient-weighted class activation mapping [8]. However, these methods mainly rely on bounding box-based detection, which provides only coarse localization and lacks the precision required for surgical navigation. Bounding boxes are inadequate for delineating organs or irregularly shaped objects, which is critical for avoiding damage during surgery. Achieving automated surgical navigation requires precise organ shape identification, as severely negative events may occur if organs are misjudged. For example, organ damage is the most common complication in surgery, including damage to the bowel, bladder, ureter, or blood vessels. In this context, AI assistance can aid novice physicians in avoiding surgical mistakes.

To address the above challenges, we developed DeepVinci, an innovative architecture for organ semantic segmentation based on a CNN, with an initial focus on gynecological surgery. Overall, our results indicate that it is possible to train a high-performance model to detect and segment organs in da Vinci surgery. DeepVinci is designed to overcome specific limitations in existing segmentation methods, including limited field of view (FoV), poor global feature representation, and ambiguous anatomical boundaries. Our architecture integrates a densely multi-scale pyramid module (DMPM), a feature fusion module (FFM), and an edge supervision network to improve segmentation performance and boundary accuracy, which is quite important for future surgical navigation and development of precision medicine.

The remainder of this article is organized as follows. Section 2 provides a brief review of semantic segmentation, efficient models, and edge supervision. Section 3 introduces the features of the dataset used in this study. Section 4 elucidates the components of the proposed DeepVinci, including the proposed densely multi-scale pyramid module (DMPM), feature fusion module (FFM), and edge supervision network. Visualized segmentation results are provided in Section 5. Finally, Section 6 presents a summary of our results.

## 2. Related Work

**Semantic Segmentation:** Building upon advances in deep learning, semantic segmentation is a fundamental task in computer vision that aims to assign pixel-level object class labels to an image. This task is a critical step in understanding a visual scene for applications such as autonomous driving, image generation, and medical diagnosis. He et al. proposed a fully convolutional network (FCN) [9], which is the ancestor of image segmentation. The FCN replaces the model output with a convolutional layer and uses upsampling to enlarge the feature map into the segmentation result. Each pixel in the image is classified to achieve pixel-wise segmentation of the image. Although the FCN can achieve end-to-end training and provide state-of-the-art performance, it does not consider the global information of the image, making it impossible to obtain detailed segmentation results. Thus, the segmentation results cannot accurately depict the contour of an object.

To overcome this lack of global context information, some researchers have applied an encoder–decoder architecture with trainable deconvolution on the decoder side. Such systems further train a better model to achieve detailed segmentation results. UNet is a well-known solution [10] based on a U-shaped network structure to obtain context and location information. The incorporated skip connection can learn multi-scale features and recover details that have been lost through downsampling. Although the encoder–decoder architecture can better record the features of the original image, it cannot draw fine contours because of its limited field of view (FoV) based on its encoder–decoder architecture.

To increase the size of the receptive field, dilated convolution was developed. Dilated convolution introduces a new parameter, namely, the dilation rate, for the convolutional layers, and the FoV of the convolution is expanded by the addition of holes so that the original 3 × 3 convolution kernel can be enlarged to 5 × 5 or even larger, eliminating the need for downsampling. However, the addition of dilated convolutions leads to additional computational complexity; thus, there is still room for further improvements in semantic segmentation methods.

**An Efficient Model:** Since the application of deep learning to computer vision, CNNs have provided the basis for image recognition and segmentation. From VGG16 [11] to ResNet [12], which introduced a residual structure, deeper networks have provided greater accuracy; however, the complexity increases accordingly. Therefore, research efforts have focused on obtaining higher accuracy for limited computing power. Researchers have found that better performance can be achieved by balancing the depth, width, and resolution of a CNN model. Thus, neural architecture search (NAS) has been adopted for designing new CNN models. For example, EfficientNet [13] was designed via NAS to achieve promising classification accuracy, lower parameter usage, and higher inference speed compared with previous CNN models. Thus, EfficientNet is a good candidate for classification applications such as semantic segmentation.

**Transformer-Based Segmentation Models:** In recent years, transformer-based architectures such as Swin Transformer [14], SegFormer [15], and SAM [16] have achieved state-of-the-art results in general segmentation tasks. These models leverage self-attention mechanisms and large-scale pretraining to learn rich global representations. Additionally, recent models like MedFormer [17], UNeXt [18], and MedSAM [19] extend transformer design into the medical domain, showing promising results in radiology and 2D/3D medical images. However, these models typically require extensive annotated data and incur high computational costs, which can limit their use in real-time intraoperative environments. In contrast, our CNN-based DeepVinci is specifically optimized for the constraints of gynecological robotic surgery, offering competitive performance without pretraining.

**Edge Supervision:** In current image segmentation approaches, edge detection is essential. To successfully separate objects from the background, the edges of objects must be accurately identified. Some well-known filtering-based techniques, e.g., Canny [20], Duba [21], and Prewitt [22], can detect changes in grayscale values to delineate the edges of an object. Medical images, such as computed tomography, X-ray, microscopic, and endoscopic images, differ from natural images because the content of medical images shows highly similar texture features. Therefore, edge supervision is essential for medical images.

DecoupleSegNets [23] is a state-of-the-art technique used to enhance object edge prediction. The authors proposed an architecture for semantic segmentation by decoupling the edges and body with different supervisions, which improved segmentation performance.

**Surgical AI Systems:** The integration of AI into surgical video analysis is an emerging trend. Trans-SVNet [24] introduces a transformer-based model for temporal reasoning in surgical videos, while Swin UNETR [25] applies Swin Transformers for volumetric segmentation tasks in clinical datasets. While powerful, these models are designed for offline analysis and require computational resources that are not feasible for edge deployment. DeepVinci, in contrast, is optimized for lightweight real-time inference and surgical applicability.

## 3. Dataset

Because of the high cost of da Vinci surgery and the technical skills required of the operator, obtaining relevant surgical videos is difficult. Thus, publicly available datasets for da Vinci gynecological surgery are limited. The ATLAS Dione dataset [5] contains videos of six da Vinci surgical tasks; however, only the tool class is annotated. The m2cai16-tool dataset [6], an endoscopic dataset with seven different surgical instruments, was released for the M2CAI 2016 Tool Presence Detection Challenge; however, no organ category is included in this dataset. In addition, the above two datasets simply use a bounding box to annotate detection targets. To address this limitation, we created a new dataset to facilitate our current study. The protocol followed in obtaining this dataset was reviewed and approved by the Institutional Review Board of Tri-Service General Hospital, Taipei, Taiwan (no. A202205062).

**Figure 1:**
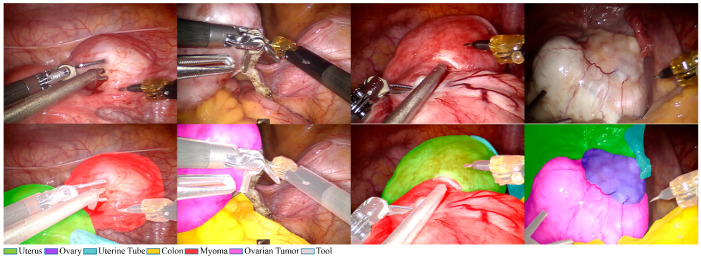
Top: Initial images. Bottom: Image annotation obtained by drawing contours of organs for the following classes: uterus (green), ovary (blue), uterine tube (cyan), colon (yellow), myoma (red), ovarian tumor (magenta), and tool (gray).

In total, we recorded 110 da Vinci surgery videos at a resolution of 1280 × 720 pixels, decomposed on a frame-by-frame basis. We removed the smoke and out-of-focus parts and randomly assigned the videos as training and testing samples to ensure that the data were independent. Surgeons annotated each image by drawing instance labels around the contours of the organs and tools. Figure 1 shows several sample images from this dataset. The dataset was divided into three parts: 1201 images for the training set, 257 images for the validation set, and 435 images for the testing set. Table 1 shows the numbers of annotated objects contained in the dataset for the uterus, ovary, uterine tube, colon, myoma, ovarian tumor, and tool. This dataset represents a major contribution to this research and is expected to facilitate applications of deep learning in da Vinci gynecological surgery.

## 4. Proposed DeepVinci

**Figure 2:**
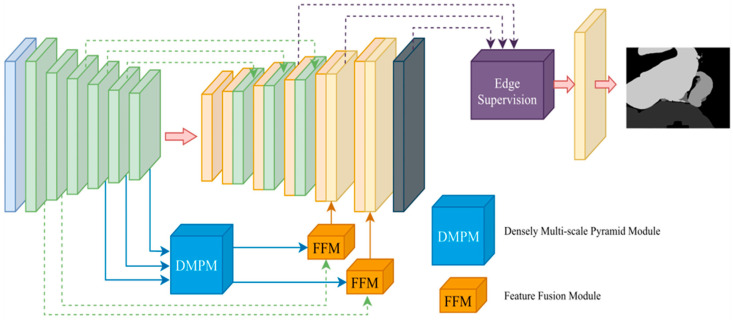
Overall framework of the proposed DeepVinci.

In this section, we introduce DeepVinci as an effective organ semantic segmentation tool. To overcome the challenges of insufficient global features and narrow FoV areas, the DeepVinci system includes the DMPM and FFM, based on EfficientNet as the backbone. To improve edge segmentation performance, the system incorporates an edge supervision network. Figure 2 shows the architecture of the entire model.

In general, segmentation results exhibit fragmentation issues when small blocks are segmented into fragmentary pieces, indicating two problems in semantic segmentation: First, the resolution of the feature map is too low, leading to inaccuracy when the image is restored to its original size. Second, the detection performance of multi-scale objects is poor. Because the system cannot focus on a large area in the image, misjudgment errors can result. These two problems are caused by the scaling of the original image through convolution and pooling, because of which the feature map gradually loses its accuracy in capturing the target. To obtain multi-scale content information, the Atrous Spatial Pyramid Pooling (ASPP) module was first proposed in DeepLabv2 [26] using a variety of dilated convolutions with different dilation rates. The dilated convolution increases the FoV of the model and retains the fine image structure information in the image. However, the ASPP module greatly increases computational complexity and memory usage, limiting its application in many tasks [27]. To overcome this problem, Wu et al. proposed a new segmentation architecture with the joint pyramid upsampling module, namely, FastFCN [28], combining the feature maps of each stage of the network and unifying them to the same size. This approach has been shown to achieve better fusion and reduce computational complexity.

In this study, we improve upon the modules mentioned above via the DMPM and FFM, which can better fuse feature maps and improve performance by using multi-scale features.

**Figure 3:**
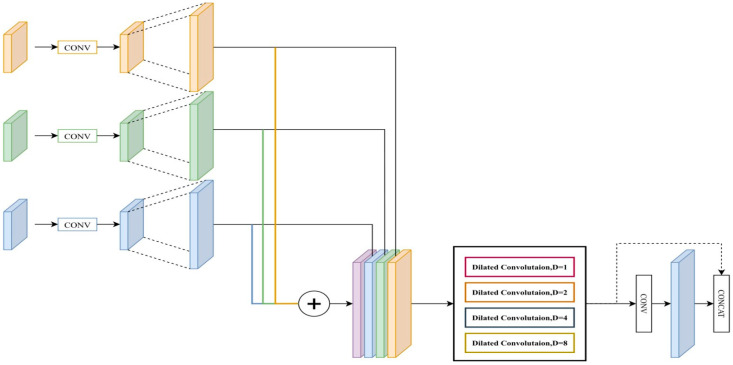
Overall architecture of the proposed DMPM. D: Dilation rate.

The DMPM, illustrated in Figure 3, is proposed to obtain diverse spatial image information using convolutions of various sizes, allowing more image details to be recorded. Combining the fused multi-scale features with dilated convolution to obtain a larger FoV can enable better feature extraction and image restoration. The DMPM first standardizes features of different sizes through convolutional layers, then captures large receptive field information via dilated convolutions, and finally refines key features using inverted residual blocks to reduce redundancy. Additionally, the subsequent FFM integrates high-level semantic features with the output of the DMPM on a pixel-by-pixel basis, further filtering out redundant information that lacks discriminative value. Therefore, the overall architecture has been thoughtfully designed to consider the redundancy issue and suppress it through the collaborative effect of the modules.

To strike a balance between computational complexity and performance, the proposed DMPM can be separated into four steps:

1. Different sizes of feature maps are unified into the same size by the convolutional layer.
2. Input feature maps are fused to generate a new map.
3. The FoV of the feature map in step 2 is enlarged via dilated convolutions with different dilation rates.
4. Inverted residual blocks are used to process low-dimensional feature spaces.

**Figure 4:**
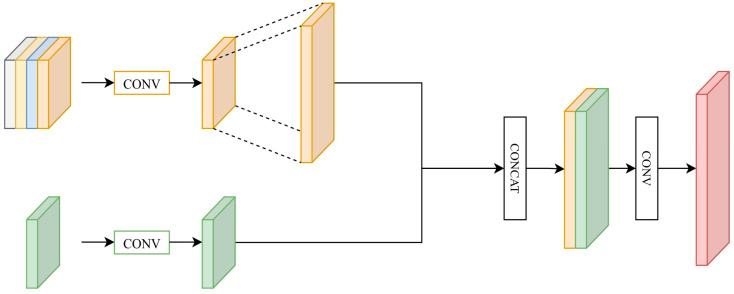
Overall framework of the proposed FFM.

The design of the FFM is shown in Figure 4. Because the DMPM generates feature maps that integrate global features, this module plays an important role in the image restoration stage. The FFM fuses the high-level feature maps from the encoding side with the DMPM output to achieve better encoding results. The FFM fuses the feature maps to obtain more global features in three steps:

1. The two input feature maps are collected into a single channel via convolution.
2. Bilinear interpolation is applied to upsample the images to the same size.
3. A pixel-wise summation operation is used to combine the feature maps.

**Figure 5:**
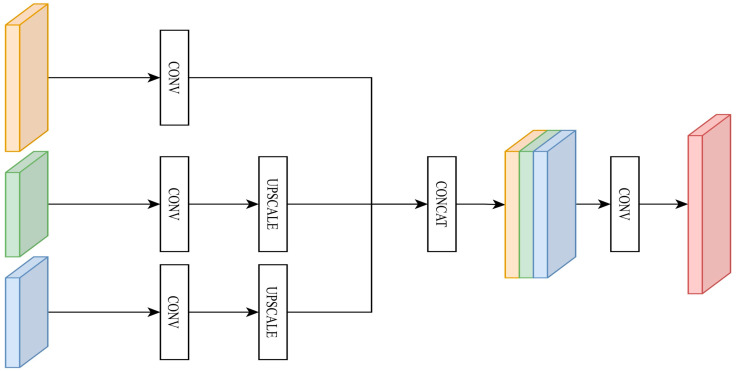
Proposed edge supervision network.

An edge supervision network is proposed to distinguish between organs and background, as shown in Figure 5. The Sobel filter is used to extract edge features from the ground truth and to fuse feature maps of different sizes through the following steps:

1. Convolution and upsampling are applied to make the feature map size consistent.
2. All of the feature maps are connected to obtain edge features.
3. A 1 × 1 convolution is used to compress the feature map dimension.

### 4.1. Optimization

In this study, we used EfficientNet-B3 as the backbone network. We performed training using the Adam optimization algorithm, with a learning rate of 0.0001, a momentum of 0.9, and a weight decay of 0.0001. The input images were resized to (480, 480), and training was conducted for 50 epochs. In terms of computational complexity, the entire DeepVinci model requires approximately 2.57 GFLOPs and 14.2 million parameters for a 480 × 480 input. Specifically, the EfficientNet-B3 backbone accounts for ~1.80 GFLOPs and ~12M parameters, while the DMPM, FFM, edge supervision network, and prediction head together contribute ~0.77 GFLOPs and ~2.2M parameters. These values highlight the model’s compactness and suitability for real-time deployment on surgical edge devices.

### 4.2. Detection Performance

We adopted a total of 435 test images for testing. We obtained the training output to evaluate the model and visualize the results. Here, the segmented masks were pasted to the original image to generate the resulting segmented images.

In this study, we applied the dice similarity coefficient (DSC) and mean pixel accuracy (MPA) to assess the performance of the model. The MPA is an evaluation metric for semantic segmentation. We determine this metric by computing the pixel accuracy for each class and then computing the average over the classes as follows:

(1) $$MPA=\frac{True\;Positive+True\;Negative}{\left(True\;Positive+True\;Negative+\;False\;Positive+False\;Negative\right)}$$

The DSC is a similar measurement that indicates the overlapping of two objects as follows:

(2) $$DSC=\frac{2*|X\cap Y|}{\left|X\right|+|Y|}$$

where X denotes the ground truth image and Y denotes the corresponding prediction of an image.

## 5. Experiments and Evaluation

In this section, we compare the proposed DeepVinci with the semantic segmentation models described in Section 2 as well as former state-of-the-art methods. For this comparison, all processes and experiments were conducted using CUDA10.1 on Ubuntu 16.0.4. The training platform had the following specifications: CPU: Intel Core i7-10700K; GPU: GEFORCE RTX 2080Ti; RAM: 128 GB.

The DSC and MPA were adopted to evaluate the performance of the methods. Because the positions of organs are more important than boundary recognition in da Vinci automated navigation surgery, we place a greater focus on the DSC, which indicates the degree of overlapping of two objects, in this study.

**Figure 6:**
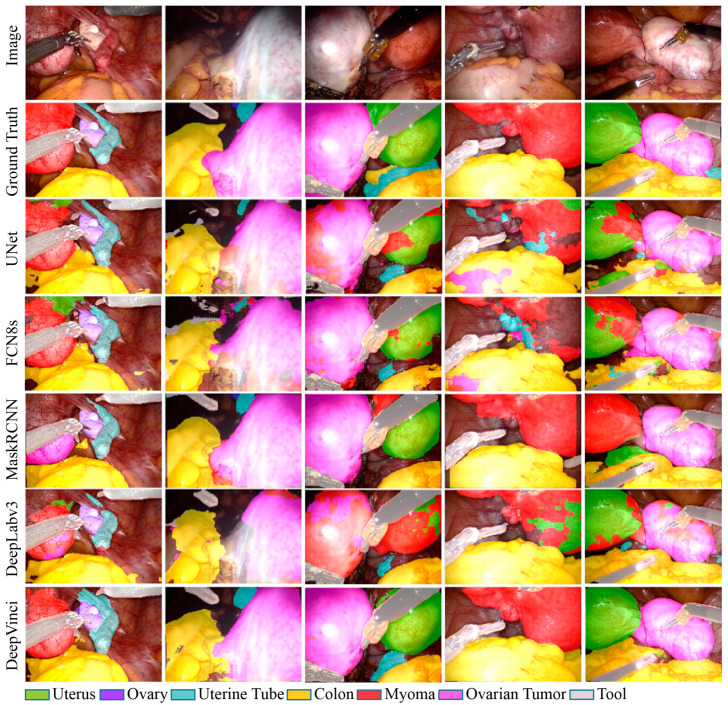
Organ detection by each model. Our DeepVinci model successfully detected the uterus, ovary, uterine tube, colon, myoma, ovarian tumor, and tool.

Table 2 shows the MPA and DSC values for various methods on the test dataset. The results show that DeepVinci can achieve good performance, with MPA and DSC values of 70.0% and 68.4%, respectively. The detection results presented in Figure 6 demonstrate that most methods achieve good performance in the tool class, as the texture of the medical tool is substantially different from that of a human organ. Because the FCN and UNet do not utilize additional methods to consider global features, the segmentation results of large targets are sometimes incomplete. In contrast, DeepLabv3 incorporates the ASPP module to increase the FoV, allowing it to focus on more global information from the image. Moreover, the regional proposal network utilized in MaskRCNN can pre-filter the organs to be detected. Thus, the performance and segmentation results of DeepLabv3 and MaskRCNN are superior to those of the FCN and UNet. Overall, the experimental results in Table 2 and Figure 6 demonstrate that DeepVinci, integrated with the proposed FFM and DMPM, can recover the feature map details lost via downsampling and achieve superior segmentation performance over the former schemes. Thus, DeepVinci presents great potential for organ semantic segmentation in da Vinci gynecological surgery.

## 6. Discussion

In recent years, da Vinci robotic surgery and AI have emerged as important developments in scientific and technological fields, and an approach for combining these two tools would enable substantial medical and technological progress. The long-term goal of our research is to establish an autopilot navigation surgery system that can provide training for less experienced surgeons and prevent mistakes during surgery. In addition, the ability to precisely identify organs or disease sites can help surgeons perform more accurate and thorough dissections of targeted tissue, while greatly relieving their burden during surgical procedures. Although it is currently difficult to integrate the proposed DeepVinci with the actual da Vinci surgery system, due to its closed system design and intellectual property protection, the proposed DeepVinci system provides a promising proof of concept for integrating AI and robotic surgery.

To the best of our knowledge, laparoscopic surgery is currently the only surgical operation that utilizes AI to help in decision-making before surgery; however, assistance is not provided in guiding the surgery [29,30]. In our graphical abstract video (see Supplementary Materials [31]), we have demonstrated the feasibility of the proposed DeepVinci system working in coordination with robotic arms. As shown in Figure 1 and Figure 6, and our abstract video (see Supplementary Materials of [31]), precise target organ segmentation and edge detection are rapidly achieved. The time delay is nearly negligible, and the system immediately provides an in situ annotation image to assist the surgeon in conducting a survey or dissection during surgery. This feature can prevent the misidentification of organs, providing a precautionary safeguard for the surgeon operating on vital tissues such as the ureter and sentinel lymph node. Thus far, we have input n = 100 patient cases into the DeepVinci system, with most of the annotated medical images being bright-field images. The proposed system achieves acceptable accuracy in distinguishing the uterus, ovary, uterine tube, colon, ovarian tumor, myoma, and robotic tool, with MPA values of 0.319, 0.566, 0.570, 0.791, 0.671, 0.822, and 0.961, respectively.

One limitation of our current model is the relatively low dice similarity coefficient (DSC) for ovary segmentation (DSC = 0.303). This can be attributed to the small size of the ovaries and their visual similarity to adjacent soft tissues, making precise boundary delineation particularly difficult under laparoscopic imaging conditions. Enhancing segmentation accuracy for such anatomically subtle and variable structures remains a challenge for automated systems.

Additionally, the model’s domain transferability is limited at this stage. All training and evaluation data were collected from a single clinical center using the same Da Vinci surgical system. Therefore, the generalizability of the model across different hospitals, imaging devices, or patient populations has yet to be demonstrated. As part of our ongoing work, we are expanding to multi-center validation and exploring domain adaptation strategies to improve robustness and real-world applicability.

Although DeepVinci demonstrates promising segmentation performance in real-world surgical settings, certain limitations remain. First, the model’s performance is currently validated on data from a single institution, which may limit its generalizability across different surgical environments. Second, segmentation accuracy for certain challenging classes (e.g., ovaries) remains suboptimal due to their small size and visual ambiguity. These issues will be addressed in our future development roadmap for the DeepVinci system.

During the removal of a primary tumor, the sentinel lymph node must be precisely located for subsequent removal to prevent metastasis. Thus, there is a pressing need for sentinel lymph node mapping via indocyanine green (ICG) fluorescence imaging. As the intensity and edge sharpness of ICG images are weak, more efforts are greatly needed to achieve better image segmentation and edge supervision.

## 7. Conclusions

In this study, we implemented and assessed DeepVinci, a high-performance encoder–decoder CNN architecture, to segment the uterus, ovary, uterine tube, colon, myoma, ovarian tumor, and medical tools in da Vinci operation images. Endoscopic videos were collected from da Vinci surgeries and were divided into training, testing, and validation sets. The proposed DMPM and FFM in DeepVinci can increase the FoV and obtain more global information, whereas the edge supervision network of DeepVinci can obtain fine edge details. Experimental results show that the proposed segmentation system can achieve DSC and MPA values of 68.4% and 70.0%, respectively, demonstrating an improvement over the former state-of-the-art method. DeepVinci shows promising results for enabling cross-validation during surgical operations by integrating segmented results with the surgeon’s judgment. Moreover, the ability of DeepVinci to perform tool identification and route tracking can enable operation training or alert triggering for cases in which a tool moves to an improper position during an operation. Building on this initial work of identifying organs in da Vinci robotic surgery, we eventually aim to develop DeepVinci as a tool for self-navigated surgical systems in the future.

## Figures and Tables

**Table 1:** Number of annotated objects and images for each class in the dataset.

	Training	Validation	Testing
Uterus	622	72	341
Ovary	502	86	129
Uterine Tube	1056	236	373
Colon	955	119	346
Myoma	845	193	344
Ovarian Tumor	384	153	246
Tool	2523	467	955
No. of Images	1201	257	435

**Table 2:** Performance comparison between prior schemes and the proposed DeepVinci scheme in terms of MPA and DSC on our dataset.

Model	Background DSC	Uterus DSC	Ovary DSC	Uterine Tube DSC	Colon DSC	Ovarian Tumor DSC	Myoma DSC	Tool DSC	Mean DSC
FCN8S	0.808	0.401	0.409	0.457	0.713	0.736	0.606	0.920	0.631
UNet	0.829	0.432	0.343	0.448	0.717	0.666	0.618	0.923	0.622
Mask RCNN	0.846	0.245	0.497	0.590	0.789	0.694	0.797	0.932	0.673
DeepLabv3	0.855	0.328	0.460	0.520	0.770	0.607	0.621	0.935	0.637
DeepVinci (proposed)	0.877	0.410	0.303	0.607	0.816	0.785	0.751	0.929	0.684
**Model**	**Background** **MPA**	**Uterus** **MPA**	**Ovary** **MPA**	**Uterine Tube** **MPA**	**Colon** **MPA**	**Ovarian Tumor** **MPA**	**Myoma** **MPA**	**Tool** **MPA**	**Mean** **MPA**
FCN8S	0.859	0.330	0.347	0.378	0.721	0.653	0.632	0.932	0.606
UNet	0.872	0.358	0.356	0.387	0.707	0.561	0.674	0.932	0.606
Mask RCNN	0.873	0.152	0.562	0.521	0.818	0.798	0.740	0.942	0.676
DeepLabv3	0.887	0.259	0.379	0.385	0.851	0.675	0.526	0.955	0.615
DeepVinci (proposed)	0.902	0.319	0.566	0.570	0.791	0.671	0.822	0.961	0.700

## Data Availability

Datasets supporting the findings of this study are available upon reasonable request. However, due to ethical considerations involving patient privacy and confidentiality, as well as intellectual property rights held by the participating institutions, complete datasets cannot be publicly shared. The Institutional Review Board (IRB) of Tri-Service General Hospital, Taipei, Taiwan (approval number: A202205062), has reviewed and approved the study protocol, specifically noting that patient confidentiality must be strictly maintained. Researchers interested in accessing the data should contact the corresponding author, Yu-Chi Wang (yuchitsgh@mail.ndmctsgh.edu.tw), to request access. Requests will be evaluated based on the intended use and the ethical considerations outlined by the IRB.
